# Cannabidiol Drugs Clinical Trial Outcomes and Adverse Effects

**DOI:** 10.3389/fphar.2020.00063

**Published:** 2020-02-25

**Authors:** Christopher S. Pauli, Matthieu Conroy, Brian D. Vanden Heuvel, Sang-Hyuck Park

**Affiliations:** ^1^ Institute of Cannabis Research, Colorado State University-Pueblo, Pueblo, CO, United States; ^2^ Department of Biology, Colorado State University-Pueblo, Pueblo, CO, United States

**Keywords:** cannabidiol (CBD), cannabidiolic acid (CBDA), cannabinoids, clinical trial, Epidiolex, hemp

## Abstract

This review aims to present completed clinical trial data surrounding the medicinal benefits and potential side effects of the increasingly popular cannabidiol (CBD)-based drug products, specifically Epidiolex. The article is divided into two sections based on if the ailment being treated by this cannabinoid is classified as either physiological or neurological conditions. In addition to describing the current status, we also examined the different primary and secondary outcomes recorded for each study, which varies greatly depending on the funding source of the clinical trial. With the recent FDA-approval of Epidiolex, this review mainly focused on trials involving this specific formulation since it is the only CBD-based drug currently available to clinicians, although all other clinically trialed CBD(A) drugs were also examined. We hope this review will help guide future research and clinical trials by providing the various outcomes measured in a single review.

## Introduction

Clinical trials inform doctors about the proven safety, efficacy, and dosage of pharmaceutical drugs through double-blind, placebo-controlled studies; however, compounds of the *Cannabis* plant are used medicinally without undergoing these trials until recently. Although 33 states have approved *Cannabis* use for medical conditions, the U.S. Drug Enforcement Administration recognizes *Cannabis* as a Schedule I drug, meaning it has no currently accepted medical use and a high potential for abuse, which also prevented research. Some cannabinoid-containing drugs could be obtained from the University of Mississippi; however, those samples are not chemically or physically representative of the legal Cannabis markets (Vergara et al., 2017). Recently, a CBD formulation, Epidiolex, was approved by the U.S. Food and Drug Administration (FDA) for two conditions, which rescheduled this formulation to a Schedule III drug, a low abuse potential classification, which allows its use in clinical trials (Drug Scheduling, n.d).

CBD is one of more than 120 naturally occurring cannabinoids found in *Cannabis sativa* L. (ElSohly and Slade, 2005; Brenneisen, 2007; Radwan et al., 2009; Fischedick et al., 2010; Andre et al., 2016; Park et al., 2019) Various CBD formulations have been tested in pre-clinical studies to have diverse medicinal properties, such as anti-nausea, anti-emetic, anti-tumor, anti-inflammatory, anti-depressant, anti-psychotic, and anti-anxiolytic; however, the variance in drug formulations used and limited sample sizes reduce the applicability of these studies in clinical applications (Costa et al., 2006; Parker et al., 2011; Micale et al., 2013; Kucerova et al., 2014; Micale et al., 2015; Bogdanović, 2017; Rock and Parker, 2017; Sumanasekera et al., 2018; Fonseca et al., 2018; Stark et al., 2019). Epidiolex (GWP42003-P) is a colorless to yellow strawberry-flavored tincture that contains 100 mg/ml of plant-derived CBD and less than 0.3% of THC(A) (Guy et al., 2014). This low-abuse and addiction potential formulation is generally well tolerated in most patients, and has a proven long-term safety profile (Schoedel et al., 2018; Laux et al., 2019). The most common side effects of Epidiolex include “somnolence; decreased appetite; diarrhea; transaminase elevations; fatigue, malaise, and asthenia; rash; insomnia, sleep disorder, and poor quality sleep; and infections”, in addition to causing mild to severe hepatic impairment in some trials (GW Biosciences, 2018; Taylor et al., 2018).

Overall, through presenting data from 16 completed clinical trials involving CBD-based drugs, we intend to inform clinicians about the current status of CBD-based drugs and guide future clinical trial designs for cannabinoid medications to have objective measurements of success, as well as detrimental side effects.

## CBD for Treating Physiological Conditions

### Epileptic Seizures

The most well-known use of Epidiolex is for its current FDA-approved indication to treat seizures associated with Lennox-Gastaut syndrome (LGS) and Dravet syndrome (DS) (GW Biosciences, 2018). Greenwich (GW) Pharmaceuticals (Cambridge, United Kingdom) is the funding source for Epidiolex's clinical trials, since they own rights to various cannabinoid formulations and delivery mechanisms patents (Dave, 2011; Flockhard et al., 2014; Guy et al., 2014; Whittle et al., 2019). For LGS particularly, 84 patients titrated Epidiolex to 20 mg/kg/day dose over 11 days and maintained that dosing for 12-weeks, which was compared to the 85 patients receiving a placebo. 43.9% of the treated patents reported improvement from baseline, which is contrasted to 21.8% reporting improvement in the placebo group. In addition to the proven efficacy, this research has shown an acceptable long-term safety profile and sustained reduction in seizures in long-term CBD treatments (Thiele et al., 2018). Furthermore, adverse effects occurred in 86% of the treated patients, with the most common reports of diarrhea (18.6%), pyrexia (12.79%), decreased appetite (12.79%), and somnolence (13.95%) (Thiele et al., 2018).

The other indication awarded to GW was specific to DS, which a similar series of clinical trials were performed following preclinical research suggesting potential seizure reduction through its agonistic action on the CB1 and CB2 receptors (Anwar et al., 2019; Silvestro et al., 2019). The same titration method, dosage, and time of use was used to evaluate the safety and efficacy on patients suffering from DS; however, this study contained a smaller participant pool of 61 patients receiving the drug and 59 receiving the placebo. The patients in the treatment group reported significant reduction in the number of seizures, with over 90% of patients reporting at least a 25% reduction in frequency and nearly 5% of patients having 100% seizure reduction. Furthermore, a decrease in the average duration of the seizure was reported in tonic-clonic, tonic, clonic, atonic, myoclonic, countable, and absence seizures; however, the placebo group also reported decreases (34%) in seizure duration as well (Guy et al., 2014; Devinsky et al., 2017).

Pre-clinical research has reported that there may be promise in patients with treatment-resistant seizure disorders, the CDKL5 deficiency disorder, or Aicardi, Dup15q, and Doose syndromes; however, due to the variability in dosage between these studies, we aim to focus only on completed clinical trial data (Devinsky et al., 2018; Szaflarski et al., 2018). Another company, INSYS Therapeutics Inc. (Phoenix, AZ), has also funded Phase 1 and 2 clinical trials to investigate a non-plant-based cannabidiol oral solution at various dosages to treat resistant seizure disorders in pediatric patients (ages 1–17). Dosages of 10, 20, and 40 mg/kg/day were provided to 20, 20, and 21 patients, respectively. This study mainly aimed to provide the pharmacokinetics of CBD, as well as the safety and dosing information; however, they did report decreases from the baseline number of seizures. Specifically, there was a reduction of tonic seizures per day and a reduction of atonic seizures per day for the respective dosing groups. While there were no serious adverse effects and non-serious adverse events were observed including anemia (10%, 25%, and 19.05%), somnolence (15%, 15%, and 33.3%), and flatulence (14.29%) in the 40 mg/kg/day dosing group (INSYS Therapeutics Inc, 2016; Parikh, 2018).

### Parkinson's Disease Tremors and Psychosis

Parkinson's disease (PD) is a progressive nervous system disease that primarily affects fine and gross movements of an individual (McKeith and Burn, 2000). Anecdotal evidence and preliminary reports documented CBD's therapeutic effect on patients (n=5) suffering movement disorders, specifically dystonia, when co-administered with each patient's traditional L-dopa medication (Consroe et al., 1986; Zuardi, 2008). However, this study used a corn oil based 150 mg dose of 99.9% pure CBD as the study drug, which differs from Epidiolex in dosage and carrier oil (Zuardi, 2008). More recently, Dr. Maureen Leehey at the University of Colorado has completed a Phase 1 trial on the safety and tolerability of Epidiolex and is currently conducting Phase 2 clinical trials. In total, 13 patients have been recruited, of which 10 have completed the clinical trial. The patients were started at 5 mg/kg/day and increased by 2.5–5 mg/kg at 3–5 day intervals to a target dose of 20 mg/kg/day, which is the GW recommended dosage. This study encompassed a wide range of primary outcomes, such as including multiple dosing levels, measuring the number of patents that left from drug intolerance, recording vital sign changes, and electrocardiogram changes, as well as preforming cognitive, anxiety, depression, movement, emotional, and sleep assessments. The secondary outcome was focused on observing any significance changes to the Movement Disorder Society-Unified Parkinson's Disease Rating Scale (MDS-UPDRS) tremor score. This study reported decreases in the total score of the MDS-UPDRS (−7.7, standard deviation = 9.4), in the Depression Short Form (−0.85, SD=3.1), in the baseline of REM Sleep Behavior Disorder Screening Questionnaire (RBDSQ), (−0.7, SD=1.8), in the Emotional and Behavioral Dyscontrol Short Form (−4.7, SD=6.1), and in the Scales for Outcomes in Parkinson's Disease (SCOPA)-Sleep-night Time Sleep score (−2.80, SD=3.91), as well as increases in the Change in Montreal Cognitive Assessment (1.0, SD=1.5) and in the Anxiety Short Form (0.33, SD=3.94), which all suggested positive outcomes. At each dosage tested, there were no serious side effects observed; however, there were reported increased diarrhea (85%), fatigue (61%) somnolence (69%), and elevated liver function tests (39%) (Leehey, 2019).

While Dr. Leehey's study suggested improvements in cognition, depression, and emotional issues associated with PD, another group investigated the anti-psychotic effects of CBD in a small group of PD patients with psychosis (n=6) (Zuardi, 2008; Zuardi et al., 2009). Zuardi's group investigated the efficacy, tolerability and safety of CBD in psychotic patients with PD through an open-label 4-week pilot study. They gave a first dose of 150 mg/day and then increased the dose by 150 mg/day each week for the remaining three weeks for a maximum dose of 600 mg/day during the fourth week. Serial neurological and physical assessments in the study found that CBD did not worsen motor symptoms or contribute any notable side effects, but it did significantly decrease psychosis symptoms (Zuardi, 2008; Zuardi et al., 2009).

### Ulcerative Colitis

Ulcerative colitis (UC), among other gastrointestinal (GI) diseases, are becoming increasingly prevalent in western countries and their etiology is still unknown (Hasenoehrl et al., 2017). With anecdotal evidence of beneficial cannabinoids for patients with GI ailments, UC was another indication that GW Pharmaceuticals investigated, and they completed Phase 2 clinical trials in 2018 (Irving et al., 2018). This multi-center study was performed in a randomized, double-blind, placebo-controlled, and parallel-group study design. Over 10 weeks, various dosages (50–250 mg) of Epidiolex were administered twice a day to 29 patients suffering from UC, and a placebo was given to 31 patients. The participants first entered into a 2-week trial to identify the maximum dose tolerance (up to 250 mg twice a day for each patient in the Epidiolex group, which they remained at that dose for the remainder of the trial. Their primary goal was to observe changes in the Mayo score (an indicator of UC severity; < 2 = remission of UC), and their secondary outcomes included measuring irritable bowel syndrome responses, UC symptom measurements, and calprotectin levels. Decreases in the Mayo score (−2.0 in treatment compared to −1.2 in control) and in the levels of calprotectin in the patients' fecal matter (−91.6, SD=295.77. in treatment; −51.3, SD=289.32 in placebo) were observed in the treatment group. There were no serious adverse effects reported by the treatment group and no mortality reported; however, 96.55% of the treatment group (76.44% of placebo group) reported some other adverse events, including dizziness (46%), somnolence (34%), and nausea (27%) (Irving et al., 2018).

## CBD for Treating Neurological Conditions

### Substance Use-Disorder

The states overwhelming support for *Cannabis* legalization has led to increases in both medical and recreational *Cannabis* use; however, an increasing number of users have reported using *Cannabis* abusively (Stinson et al., 2006; Agrawal et al., 2014). For these patients, Epidiolex was proposed to reduce *Cannabis* use, and improve the quality of life of the patients while abstaining from *Cannabis*. McLean Hospital funded a Stage 1 pilot feasibility study to evaluate Epidiolex as a pharmacotherapy for *Cannabis* use disorder suffering adults. They recruited 10 participants for this randomized trial, which half were given up to 800 mg of Epidiolex over 6 weeks, and the other half were given a placebo. One of the patients in the CBD arm was excluded for poor medication adherence; however, with the limited remaining participants that completed the study (n=4), they reported an increase in the number of inhalations of *Cannabis* per day for the CBD arm, suggesting that Epidiolex increased *Cannabis* use. Furthermore, the secondary outcomes of this study will measure “treatment retention, patient satisfaction, *Cannabis* withdrawal, cannabis craving, depressive symptoms, anxiety symptoms, compliance, and cigarette use”; however, none of these results have been reported (Hill, 2016).

Although the study on treating *Cannabis* use disorder results are still unclear, another group is investigating the potential of CBD to reduce opioid cravings after observing successful selective inhibition of drug-seeking behaviors in rats (Gonzalez-Cuevas et al., 2018). This research inspired a clinical trial led by Dr. Yasmin Hurd of the Icahn School of Medicine at Mount Sinai to sponsor research into measuring CBD's efficacy to inhibit in- and out-clinic cravings for heroin-dependent humans. The study administered CBD four times to patients that have abstained heroin use for at least 6 days prior to the first session, in one of two dosages, either 400 or 800 mg. Due to the small sample size (n=10), the two different dosage arms were combined to determine the efficacy in reducing cravings, which they reported a reduction of both in-clinic and out-clinic cravings; however, a larger sample size is needed to form more definitive conclusions. Additionally, no serious adverse effects were reported, and 4 of 6 participants reported non-serious adverse effects including diarrhea, drowsiness, increased appetite, change in urine color, and feeling down or irritable (Hurd, 2013).

### Cognitive Dysfunction in Schizophrenia

CBD has also been examined as a potential treatment for cognitive dysfunction in schizophrenic patients. A single clinical study on CBD's effects on schizophrenia has been completed by Dr. Mohini Ranganathan from Yale University in 2013. This was a 6-week, randomized, placebo-controlled, parallel group, fixed-dose study of oral CBD (600 mg/day) in 36 stable antipsychotic-treated patients diagnosed with chronic schizophrenia. There was a significant decrease in the total score of the Positive and Negative Syndrome Scale (PANSS), which suggests a decrease in overall symptoms; however, there was no significant drug × time interaction, which means the effect of treatment did not depend on time. Conversely, in their secondary outcome measurement, there was no significant effect on the Measurement and Treatment Research to Improve Cognition in Schizophrenia (MATRICS) Consensus Cognitive Battery composite score, which evaluates key cognitive functions in schizophrenic patients, but there was a significant drug × time interaction. There were no serious adverse side effects observed in this study, and the only side effect observed in the CBD group compared to the placebo was sedation (Boggs et al., 2018).

## Summary Table of Examined Conditions

To summarize the study sample sizes, dosing regiments, and effectiveness of using CBD-based drugs as a treatment option, we have created a table to describe the results and provided their sources to be able to further investigate the topics that have been discussed (**Table 1**).

**Table 1:** Clinical trials results from epidiolex-focused studies.

Condition	Drug Dosage	% Symptom Reduction	% Adverse Effects	Study Size	Citation
Lennox-Gastaut Syndrome	20 mg/kg/day, titrated over 11 days	43.9%	86%	n = 156	Thiele et al., 2018
Dravet Syndrome	20 mg/kg/day, titrated over 11 days	43%	93%	n = 108	Devinsky et al., 2017
Parkinson’s Disease	20 mg/kg/day, titrated by 2.5–5 mg/kg at 3–5 day intervals	NA	100%	n = 13	Leehey, 2019
Ulcerative Colitis	50–200 mg/day	28%	96.6%	n = 60	Irving et al., 2018
Cannabis Use Disorder	Up to 800 mg/day	0%	NA	n = 4	Hill, 2016
Opioid Use Disorder	400 mg/day or 800 mg/day	100%	66.7%	n = 10	Gonzalez-Cuevas et al., 2018
Cognitive Dysfunction	600 mg/day	0%	71.4%	n = 36	Boggs et al., 2018

Clinical trial results from Epidiolex-focused studies: The table summarizes the results from the currently completed clinical trials that have tested Epidiolex’s efficacy against various conditions. The % symptom reduction is reported as the percentage of patients in the treatment group to report improvement in the treatment of their condition. Similarly, the % of adverse effects refers to the total percentage of treatment group patients that reported any adverse effect. The sample size describes the number of patients that have completed the clinical trial currently.

## Conclusions

While this review's focus is on the current uses of Epidiolex and similar formulations, there is a wide-spread issue of private companies forming nutraceutical blends of CBD with various carrier oils, terpene mixtures, and additives that are being used to treat various conditions without any clinical evaluation. While the main ingredient, CBD, has been proven numerous times to be well-tolerated in a wide range of patients suffering from various ailments, there still presents a public danger of unregulated products being distributed without any clinical information about these mixtures.

From comparing the various clinical trials on CBD, there are also clear incongruencies that need to be addressed in the clinical trial design process. The differences in primary and secondary outcome measurements becomes concerning since there is a lack of comparability that is needed for widely applicable drug-safety. For example, Dr. Leehey's study evaluated more parameters and collected more comprehensive patient data than the more positive-focused outcomes observed in the GW Pharmaceutical funded studies, as well as using different exclusion criteria. The physical and psychological measurements such as those in Dr. Leehey's study could act as a model study design for future clinical trials of Epidiolex to ensure safety and efficacy of new CBD-containing drug formulations. Furthermore, it's notable that nearly 90% of patients in Dr. Leehey's study had diarrhea; whereas, this wasn't reported in other Epidiolex studies, which suggests either PD patients have a negative GI response to Epidiolex, or there are potential inconsistencies in the formulations being tested. The latter is supported by the GW Pharmaceutical Epidiolex drug pamphlet, which describes the formulation as “a clear, colorless to yellow liquid”. This color variation could represent a change in chemical composition of one or more of the ingredients that may cause the outcome variations observed between clinical trials (GW Biosciences, 2018).

While the study designs are inconsistent, the drug and dosage being examined also varies significantly between studies. While Epidiolex has a concentration of 100 mg/ml and a maximum recommended dose of 20 mg/kg/day, some studies examined drastically deviated from that recommendation (GW Biosciences, 2018). The study performed to examine CBD's efficacy on UC patients used 1–5 50 mg capsules; whereas, the dosage ranged from 400 mg to 800 mg doses in studies for drug-cessation (Hurd, 2013; Hill, 2016; Irving et al., 2018). This could explain the differences in the side effects of each study; however, further work is needed to conclude if the variance was due to CBD concentration or delivery method. Other studies, such as the Yale's study into cognitive decline used a fixed-dose of 600 mg of “active cannabidiol”, but did not specify delivery mechanism in their clinical trial results, which makes it difficult to compare the results to other clinical trials (Boggs et al., 2018). Along this note, it should be required to describe in detail how the dose was given since some studies had specified they increasingly titrated the dose of CBD, which allows the patient to build a tolerance to the drug before receiving the maximum dose (GW Biosciences, 2018).

Another issue observed while reviewing these clinical trials is that they are privately funded by the company that developed the drug. Thus, there is a financial interest tied to this drug being approved, which may have directed the primary and secondary outcome measurements to be focused on more positive effects. This can be supported through comparing studies lead by GW Pharmaceuticals to Dr. Leehley's at the University of Colorado, since Dr. Leehley's had examined more potential negative effects that GW did not include (Devinsky et al., 2017; Thiele et al., 2018; Leehey, 2019). This is compounded by the vast exclusion criteria, and limited inclusion criteria on these studies, since patients that meet the excluded criteria may not necessarily be excluded from using the approved drug, which can be dangerous for public safety to generalize the safety of a drug based on data about a small homogeneous sample size. While these improvements would better the clinical trial process, the main aim of this review is to inform doctors, patients, and scientists of the clinical trials currently completed, and help guide future trials to ensure the safest drugs possible.

## Author Contributions

S-HP was the primary investigator to this research, was responsible for the initial draft, and conceptualized the idea for this review. CP prepared the manuscript. S-HP and CP edited and completed the manuscript. MC assisted in reviewing literature surrounding this subject. S-HP and BH funded this research.

## Funding

This research was funded solely through the Institute of Cannabis Research at Colorado State University – Pueblo.

## Conflict of Interest

The authors declare that the research was conducted in the absence of any commercial or financial relationships that could be construed as a potential conflict of interest.
